# Outcomes after fixation of rib fractures sustained during cardiopulmonary resuscitation: A retrospective single center analysis

**DOI:** 10.3389/fsurg.2023.1120399

**Published:** 2023-01-23

**Authors:** Nicole Maria van Veelen, Lea Buenter, Valérie Kremo, Jesse Peek, Alfred Leiser, Peter Kestenholz, Reto Babst, Frank Joseph Paulus Beeres, Fabrizio Minervini

**Affiliations:** ^1^ Department of Orthopedic and Trauma Surgery, Lucerne Cantonal Hospital, Lucerne, Switzerland; ^2^ Department of Thoracic Surgery, Lucerne Cantonal Hospital, Lucerne, Switzerland; ^3^ Department of Health Science and Medicine, University of Lucerne, Lucerne, Switzerland

**Keywords:** rib fracture, resuscitation, rib fixation, flail chest, rib stabilization

## Abstract

**Background:**

Historically rib fractures have been typically treated non-operatively. Recent studies showed promising results after osteosynthesis of rib fractures in trauma patients with flail segments or multiple rib fractures. However, there is a paucity of data on rib fixation after cardiopulmonary resuscitation (CPR). This study evaluated the outcomes of patients who received rib fixation after CPR.

**Methods:**

Adult patients who received surgical fixation of rib fractures sustained during CPR between 2010 and 2020 were eligible for inclusion in this retrospective study. Outcome measures included complications, quality of life (EQ 5D 5L) and level of dyspnea.

**Results:**

Nineteen patients were included with a mean age of 66.8 years. The mean number of fractured ribs was ten, seven patients additionally had a sternum fracture. Pneumonia occurred in 15 patients (74%), of which 13 were diagnosed preoperatively and 2 post-operatively. Six patients developed a postoperative pneumothorax, none of which required revision surgery. One patient showed persistent flail chest after rib fixation and required additional fixation of a concomitant sternum fracture. One infection of the surgical site of sternal plate occurred, while no further surgery related complications were reported. Mean EQ-5D-5L was 0.908 and the average EQ VAS was 80. One patient reported persisting dyspnea.

**Conclusion:**

To date, this is the largest reported cohort of patients who received rib fixation for fractures sustained during CPR. No complications associated with rib fixation were reported whereas one infection after sternal fixation did occur. Current follow-up demonstrated a good long-term quality of life after fixation, warranting further studies on this topic. Deeper knowledge on this subject would be beneficial for a wide spectrum of physicians.

## Introduction

Rib fractures are a very common complication after cardiopulmonary resuscitation (CPR). Several studies have shown rib fractures to occur in between 26%–80% of patients who underwent CPR for reasons other than trauma with most patients fracturing more than six ribs (1–3). These patients are at a high risk for pulmonary complications such as pneumonia, acute respiratory distress syndrome (ARDS), pneumothorax and empyema, as the risk increases in correlation to the number of fractured ribs (4).

In the past, rib fractures were treated conservatively with a combination of adequate analgesia, respiratory support, and aggressive pulmonary toilet (5). Currently, surgical treatment of flail chest sustained from blunt trauma have been increasingly performed in selected patients with improved results in regard to mortality, duration of mechanical ventilation, length of stay in hospital, incidence of pneumonia and tracheostomy when compared to conservative treatment (5–7). However, literature on rib fixation after cardiopulmonary resuscitation is scarce and it is therefore unclear whether these patients would have the same benefit as those after blunt trauma. Patients who sustained their injury during cardiopulmonary resuscitation have underlying conditions which caused the cardiac arrest, potentially affecting the outcome of this population. The comorbidities may increase the risk of surgical complications but at the same time may cause these patients to be more prone to pulmonary complications (8).

Therefore, the primary aim of this study is to assess the in-hospital outcome and long-term quality of life of patients receiving rib fixation after cardiopulmonary resuscitation. Furthermore, the surgery characteristics were evaluated.

## Patients and methods

This study was approved by the Medical Ethical Review Board (EKNZ 2019-00618) and was performed in accordance with the Declaration of Helsinki. It was designed as a retrospective cohort study with an additional prospective follow-up by telephone interview. Informed consent was obtained from the patients approached to obtain current follow-up. The need for consent for the use of the retrospective data was waived by the Institutional Review Board.

All patients >18 years of age who underwent rib fixation for flail chest or multiple rib fractures between 2010 and 2021 in a level-1 trauma center were screened for eligibility. Only patients who sustained their rib fractures as a result of cardiopulmonary resuscitation were eligible for inclusion. Data were collected from the institution`s electronic patient records. Collected data included demographics (age, sex), American Society of Anesthesiologists (ASA) score, pre-existing comorbidities [asthma, chronic obstructive pulmonary disease (COPD), congestive heart failure, hypertension, diabetes mellitus], and smoking status. Patients were considered smokers if they were smokers at any time in life.

In addition, the following concomitant findings were documented: pneumothorax, tension pneumothorax, hemothorax, pulmonary contusion, sternum fracture.

Fracture-related characteristics and the presence of radiological signs of aspiration were evaluated by computer tomography (CT) imaging. The characteristics evaluated were total number of fractured ribs, level of rib fractures, presence of bilateral fractures, displaced fractures, dorsal fractures, and the presence of a flail chest. The level of fracture was divided into three groups: upper (costa 1–4), middle (costa 5–9) and lower (costa 10–12). Fractures with a displacement of one shaft width or more were defined as displaced (9). Fractures dorsal to the midaxillary line were defined as dorsal fractures. Flail chest was diagnosed based on CT imaging if three or more consecutive ribs were fractured in at least two places.

The in-hospital outcomes included the length of hospital stay, intensive care unit (ICU) length of stay, need for mechanical ventilation, need for tracheostomy and mortality during the index hospital stay. Furthermore, the occurrence of pulmonary complications [pneumonia, acute respiratory distress syndrome (ARDS), postoperative pneumothorax], and surgery related complications (surgical site infection, implant failure, removal of implant, nonunion/malunion, revision surgery) were evaluated. Implant failure was defined as breakage or dislocation of an implant. ARDS was defined by severe hypoxemia with a PaO2/FiO2 ratio less than or equal to 100 mmHg. Pneumonia was defined by the appearance of clinical signs and symptoms (temperature >38.5 degrees Celsius, coughing, and decreased oxygen saturation) requiring antimicrobial therapy, with or without positive mucus cultures. The surgery related evaluated characteristics were the surgical approach (anterior, antero-lateral, posterior, posterior-lateral, combined), duration of surgery in minutes, number of ribs fixed, type of implant (plate or splint), bilateral fixation. In case of multiple surgeries, surgery-related characteristics of each surgery were collected individually.

An attempt was made to contact all patients by phone to assess their current quality of life and the occurrence of dyspnea. The quality of life was assessed using the EQ-5D-5L questionnaire and the occurrence of dyspnea with the Modified Medical Research Council (mMRC) dyspnea scale. The EQ-5D-5L consists of a descriptive system and the EQ Visual Analog Scale (EQ VAS). The descriptive system measures the patients' health using five levels of severity (ranging from no problems to extreme problems) in the following five dimensions: mobility, self-care, usual activities, pain/discomfort, and anxiety/depression.

An index was calculated comparing the patient's data to an index population. Since there is no data for the Swiss population, the German one was chosen as a reference (10). As all included patients were from the German-speaking area of Switzerland, the authors believe that from the possible choices, the German population best represents the current cohort. The maximum score is 1.000. The EQ VAS assesses general health on a scale from 0 to 100, where zero represents worst possible and 100 best possible general health. The mMRC dyspnea scale ranges from zero to four, with zero representing dyspnea only when performing a heavy effort and four representing dyspnea while getting dressed/not able to leave the house.

### Surgery characteristics

The surgery-related characteristics are presented in Table 1. All surgeries were performed using the MatrixRIB Fixation System (Depuy Synthes, Oberdorf, Switzerland). One patient received two separate surgeries three days apart making the total number of surgeries 20. The majority of the patients (95%) underwent rib fixation due to mechanical instability of the thorax with either paradox breathing or inability to wean the patient from ventilation. The remaining case was performed at the end of an urgent thoracotomy due to a hemodynamically relevant hemothorax. The average duration of surgery was 176 min (range 77–402 min). The anterior approach was the most commonly used (45%). The mean number of ribs fixed per surgery was five. Out of the 17 patients with bilateral fractures, 13 (76%) received bilateral fixation. One of these patients had fixation of each side on a different day (two separate surgeries). Plates were used in all patients and two patients received a splint. Seven patients had a concomitant sternum fracture, all of which were stabilized with a plate. Two patients required a revision surgery. In the first case revision surgery was performed due to an infection of a sternum osteosynthesis which had been performed in the same setting as the rib fixation. The second patient had an additional fixation of a sternal fracture in a separate operation setting as the thorax had remained unstable despite rib fixation. No other surgery related complications were reported.

**Table 1:** Surgery characteristics.

Characteristics	*n* = 20
Surgical approach, *n* (%)	
Anterior	9 (45)
Anterolateral	4 (20)
Combination	7 (35)
Number of ribs fixed, mean ± SD	4.7 ± 1.9
Complications	
Infection	1 (5)^a^
Implant failure	0 (0)
Nonunion/malunion	0 (0)
Removal of implant	0 (0)

SD, standard deviation.

^a^ Infection of sternal implant.

## Results

### Patients and injury characteristics

The baseline characteristics are presented in Table 2. Nineteen patients met the inclusion criteria. Of those, seventeen patients (89%) were male. The average age was 66.8 years. All patients had an ASA score >3. Cardiac arrest leading to the need for resuscitation had a cardiac etiology such as arrhythmia or myocardial infarction in fourteen patients. Two patients had central pulmonary embolisms which had led to cardiac arrest, one patient had severe hyperkalemia and one patient suffered massive hemorrhage from a carcinoma located in the hypopharynx. In one patient the cause of cardiac arrest remained unknown. Duration of cardiopulmonary resuscitation was known in 15 patients and ranged from 11 to 60 min (mean 25 min).

**Table 2:** Baseline characteristics.

Characteristics	*n* = 19
Age, mean ± SD	66.8 ± 6.8
Male sex, *n* (%)	17 (89)
ASA class, *n* (%)	
1–2	0 (0)
>2	19 (100)
Smoker, *n* (%)^a^	8 (73)
Comorbidities	
Asthma	0 (0)
COPD	4 (21)
Diabetes	2 (10)
Heart failure	4 (36)
Hypertension	7 (36)
Number of ribs fractured, mean ± SD	9.8 ± 3.3
Bilateral rib fractures, *n* (%)	17 (89)
Level rib fractures, *n* (%)	
Upper	19 (100)
Middle	18 (95)
Lower	1 (5)
Displacement, *n* (%)	15 (79)
Dorsal fractures, *n* (%)	3 (16)
Flail segment, *n* (%)	7 (37)
Concomitant findings, *n* (%)	
Radiological signs of Aspiration	8 (42)
Hemothorax	6 (32)
Pneumothorax	4 (21)
Pulmonary contusion	1 (5)
Sternum fracture	7 (36)

ASA, American Society of Anesthesiologists; BMI, body mass index; COPD, chronic obstructive pulmonary disease; SD, standard deviation.

^a^ Information missing for 10 patients.

**Figure 1:**
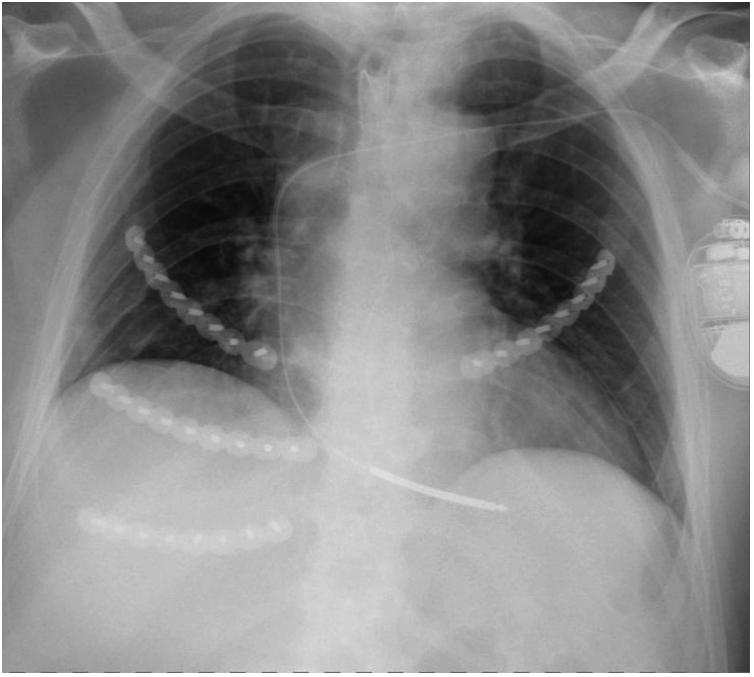
Example of bilateral plating of rib fractures sustained during CPR.

The mean number of ribs fractured was 10 (±3). Seventeen patients (89%) had bilateral rib fractures (Figure 1) All but one fracture were in the upper and the middle level, with 100% of the patients having involvement of the upper level. Fifteen patients (79%) had displaced fractures, only three (16%) had dorsal fractures. Seven patients (37%) had a flail chest. The most common concomitant finding was a sternum fracture which was present in seven patients (36%). Six patients (32%) had a hemothorax and four patients (21%) a pneumothorax. One patient (5%) had a pulmonary contusion. There were radiological signs of aspiration after cardiopulmonary resuscitation in eight patients (42%).

### In-hospital outcomes

The outcomes are summarized in Table 3. The mean length of stay in hospital was 24.9 days. All patients were admitted to the ICU and required mechanical ventilation. The average length of mechanical ventilation was 12.1 days. For the patients who remained ventilated from reanimation to surgery, the mean time between rib fixation and end of mechanical ventilation was three days (range 1–7). The mean time between CPR and rib fixation was 8.5 days (range 0–17). On average, 15.6 days were spent in the ICU and out of these on average nine days (range 1–27 days) were postoperative. One patient was discharged from the ICU to the normal ward prior to rib fixation. Eight patients (42%) received a tracheostomy. Pneumonia was diagnosed in fifteen patients (79%) of which 13 (86%) occurred preoperatively. Six patients (32%) developed a pneumothorax after surgery. ARDS was diagnosed in two patients after resuscitation but before surgery, no further cases were diagnosed after rib fixation. None of the patients died during surgery. Five patients (26%) died after surgery during the hospital stay, however the causes of death were not directly related to the surgery or ventilation. According to their presumed will, these patients had been put on palliative care as they showed no signs of neurological recovery during their further course.

**Table 3:** In-hospital outcomes.

Outcome	*n* = 19
Length of stay in days, mean ± SD	
Hospital	24.9 ± 10.8
Intensive care	15.6 ± 9.6
Intensive care unit admission, *n* (%)	19 (100)
Need for mechanical ventilation, *n* (%)	19 (100)
Duration of mechanical ventilation in days, mean ± SD	12.1 ± 9.4
Duration of mechanical ventilation after fixation, mean ± SD (8 patients)	3 ± 2.5
Time to fixation in days, mean ± SD	8.5 ± 5.7
Tracheostomy, *n* (%)	8 (42)
Pneumonia, *n* (%)	15 (79)
Post CPR	13 (68)
Postoperative	2 (11)
Pneumothorax postoperative, *n* (%)	6 (32)
ARDS in context of CPR, *n* (%)	2 (11)
Mortality, *n* (%)	5 (26)

ARDS, acute respiratory distress syndrome; CPR, cardiopulmonary resuscitation; SD, standard deviation.

### Current follow-up

Nine patients were able to be interviewed by phone. One patient was not able to provide any information due to persistent neurological impairment and one patient refused the interview. The remaining three had deceased since being discharged from hospital, the cause of death is unknown as they were not readmitted to the study hospital. The median time between rib fixation and telephone follow-up was 21 months (range 5 to 49 months). The mean score for the EQ-5D-5L was 0.903 (range 0.506–1.000). The average score on the EQ VAS was 80.6 (range 50 to 100). One patient suffered from severe dyspnea according to the mMRC scale; none of the other patients reported having difficulty breathing. None of the patients needed implant removal or experienced any other complications.

## Discussion

To date this is the largest published series of patients who underwent fixation of rib fractures which were sustained during cardiopulmonary resuscitation. Due to the limited literature available, there is a lack of information about the indication and outcomes of fixation of such fractures, even though rib and sternum fractures are the most common injuries after cardiopulmonary resuscitation (11).

Rib fixation in patients with multiple rib fractures or flail chest after blunt thoracic trauma has been reported as a safe procedure with good quality of life at long-term follow up when compared to a reference population (12, 13). Furthermore, a recent meta-analysis showed a reduction of mortality for patients with flail chest who underwent rib fixation, along with a reduction of ICU length of stay, duration of mechanical ventilation and pneumonia rate (7, 14). Patients with multiple rib fractures or a flail chest after cardiopulmonary resuscitation would likely also benefit from rib fixation especially considering that many of these patients have preexisting medical issues which could negatively influence recovery from multiple rib fractures. Kocher and coll. performed an analysis on rib fixation for ventilator-dependent patients with traumatic flail chest including seven patients who sustained their injury during CPR. No in-depth subgroup analysis of these patients was performed, however the authors mentioned that comorbidities prolonged patients' weaning more than expected (15).

Four case reports have previously been published each describing one to four patients who underwent rib fixation after CPR. The majority of these cases had bilateral fractures and all patients were male. The total number of fractures ranged from 2 to 13 with the number ribs fixed ranging from two to six. Half of the patients had bilateral fixation (16–19). These cases are in line with the cohort reported in this study, where similarly most of the patients were male, the mean number of ribs fractures was ten and 89% had bilateral fractures. The average time to fixation was shorter in the current study than in the cases previously reported [8.5 days vs. 15.6 (9–26) days]. A recent study performed a comparison of 13 patients with and ten patients without fixation for rib fractures sustained during CPR. In this study only patients who had undergone emergency coronary angiography with clinical and radiological flail chest, and a minimum ICU stay of 5 days were included. The study was unable to demonstrate a significant difference regarding the duration of ventilatory support between the groups, however when only the duration of ventilation after fixation was compared with the non-operative group there was a significant difference (20). As the inclusion criteria were very limiting, both groups of the study were small, and the results may not apply for other patients after CPR. The duration of mechanical ventilation for the patients included in this current study was three days shorter than those with rib fixation included in the study by Dorn et al. This could be due to different hospital policies but may also be caused by the exclusion of patients with an ICU stay shorter than five days or those with a non-cardiac reason for CPR. Compared to the patients who did not undergo rib fixation included in the study by Dorn et al. the duration of ventilation after fixation in the current study was 14 days shorter, supporting the assumption that the patients may benefit from rib fixation.

The patients in this study had a very high score in the EQ-5D-5L with a mean index of 0.903, which is higher than the general elderly (65 years) reference population of Germany (mean 0.84) (10). In patients receiving rib fixation after blunt thoracic trauma the mean EQ-5D-5L index was lower than for the patients in this study with 0.85 for patients with flail chest and 0.79 for patients with multiple rib fractures (12). Similarly, the EQ VAS in this cohort was similar to the general elderly reference population for 65 to 69-year-olds (80 vs. 83) and slightly higher than the reference score for 70 to 74-year-olds (80 vs. 74) (10). The reason for this high quality of life remains unclear. Interestingly, a study on the quality of life after cardiac arrest in Switzerland, in which however the occurrence and treatment of rib fractures was not mentioned, showed a similarly high EQ VAS of 87.5 (21). A further study which evaluated the quality of life after cardiac arrest with the SF-12 questionnaire showed patients scored higher than the reference population for the mental components and approximately the same for physical components (22). It therefore remains unknown, whether rib fixation influenced the quality of life in the patients included in this study or if patients generally score higher in quality of life questionnaires after successful cardiopulmonary resuscitation.

Two patients in this cohort required revision surgery, however none of the revisions were directly associated with the rib fixation (one infection of sternal osteosynthesis and one delayed additional sternal osteosynthesis). No other surgery related complications (infection of rib osteosynthesis, implant failure, removal of implant, nonunion) were noted, whereas Peek et al. reported implant or surgery related complications in 10.3% (14). This discrepancy is likely due to the small number of patients in this cohort although previously published case reports of rib fixation after cardiopulmonary resuscitation also showed no surgery related complications (16–18).

Sternal fractures have been reported to occur in 8%–55% of patients after CPR. In the current cohort 36% of the patients had concomitant sternal fractures (1–3). All sternum fractures in this study were stabilized. To date guidelines regarding patient selection for surgical fixation of sternal fractures are lacking. Possible indications include displacement, respiratory insufficiency, ventilator dependence, severe pain and non-union (23, 24). For the patients included in this study, the decision to stabilize the sternum was made by the treating surgeons and was based on chest wall instability. One patient, who did not have sternal fixation during the rib fixation surgery required fixation at a later date due to persistent chest wall instability. Despite the lack of high quality literature, this seems to support the decision to stabilize the sternum fractures in this cohort.

This study reported pneumonia in 79% of the patients, postoperative pneumothorax in 32% and ARDS in 11% (all preoperative). No information on these outcomes was found in the previously published case reports (16–19). In patients with traumatic rib fractures pulmonary complications seem to be less frequent with pneumonia reported in 17.9%, pneumothorax in 2.2% and ARDS in 2.6% (14). Similarly, more patients in this study required tracheostomy than has been reported in trauma patients (42% vs. 15%) (14).

The higher rate of pulmonary complications is most likely due to the high incidence of pneumonia after cardiopulmonary resuscitation, which is reported to be as high as 50% (25). Furthermore, a recent study was able to demonstrate an increased risk of pneumonia in patients after CPR when multiple rib fractures were present (26). If the cases in which pneumonia occurred preoperatively (13 out of 15 cases) as a complication of the cardiopulmonary resuscitation are disregarded, the frequency (11%) is very similar to that reported for trauma patients. Patients experiencing cardiac arrest are at risk for lung injury including ARDS and pneumonia due to a variety of mechanisms, including emesis, which occurs in 30% of the patients around the time of out-of-hospital cardiac arrest, implying a high risk for aspiration and pneumonia (27). This study supports these findings as there were radiological signs of aspiration in 42% of the patients and 11% developed ARDS. The development of and recovery from pneumonia and ARDS after cardiopulmonary resuscitation would be an interesting topic for a larger study to investigate as rib fixation may influence the course of illness. Furthermore, the mean age of the patients in this cohort was 13.8 years older than the patients included in the study by Peek et al. which could negatively influence the occurrence of pulmonary complications (14).

All patients in this study and in the previously published case reports needed ICU admission. Only Claydon et al. reported length of stay in the ICU for his four patients. With a mean of 37.8 days, it was more than twice as long as for the patients in our cohort (16 days) (18). Due to the small number of patients in both groups this difference cannot be explained and may be caused by local variations in standards of care or patient comorbidities. Other authors have reported the length of ICU stay after cardiac arrest to range from five to twelve days (28, 29). The wide range is most likely due to different local standards (one study from the UK and one from Austria) and possibly also due to variations in study population. A conclusion as to why the length of ICU stay in this current cohort was longer can therefore not be drawn.

There are some limitations to this study that must be mentioned. The first limitation is the small number of patients included. This limits the ability to draw any conclusions and permits only the recognition of trends. The second and main limitation is the lack of comparison to a similar cohort without rib fixation. This would be a very interesting approach for further studies and would elucidate the advantages and disadvantages of rib fixation after successful cardiopulmonary resuscitation. A recent study attempted to perform such a comparison but was limited by the small number of patients included and the restricting inclusion criteria (20).

## Conclusion

This study showed good long-term quality of life and acceptable morbidity after fixation of rib fractures sustained during cardiopulmonary resuscitation. These promising results warrant further studies with a larger number of patients along with a comparison to patients treated non-operatively. Further knowledge of this subject would be beneficial for a wide spectrum of physicians including thoracic surgeons, intensive care physicians and trauma surgeons.

## Data Availability

The raw data supporting the conclusions of this article will be made available by the authors, without undue reservation.
